# Mechanism in External Field-mediated Trapping of Bacteria Sensitive to Nanoscale Surface Chemical Structure

**DOI:** 10.1038/s41598-017-15086-1

**Published:** 2017-11-30

**Authors:** Shiho Tokonami, Emi Shimizu, Mamoru Tamura, Takuya Iida

**Affiliations:** 1 Department of Applied Chemistry, Graduate School of Engineering, Osaka Prefecture University, 1-2, Gakuencho, Nakaku, Sakai, Osaka, 599-8570 Japan; 2 Department of Physical Science, Graduate School of Science, Osaka Prefecture University, Sakai, Osaka, 599-8570 Japan; 3 Research Institute for Light-induced Acceleration System (RILACS), Osaka Prefecture University, 1-2 Gakuen-cho, Naka-ku, Sakai, Osaka, 599-8570 Japan

## Abstract

Molecular imprinting technique enables the selective binding of nanoscale target molecules to a polymer film, within which their chemical structure is transcribed. Here, we report the successful production of mixed bacterial imprinted film (BIF) from several food poisoning bacteria by the simultaneous imprinting of their nanoscale surface chemical structures (SCS), and provide highly selective trapping of original micron-scale bacteria used in the production process of mixed BIF even for multiple kinds of bacteria in real samples. Particularly, we reveal the rapid specific identification of *E. coli* group serotypes (*O157:H7* and *O26:H11*) using an alternating electric field and a quartz crystal microbalance. Furthermore, we have performed the detailed physicochemical analysis of the specific binding of SCS and molecular recognition sites (MRS) based on the dynamic Monte Carlo method under taking into account the electromagnetic interaction. The dielectrophoretic selective trapping greatly depends on change in SCS of bacteria damaged by thermal treatment, ultraviolet irradiation, or antibiotic drugs, which can be well explained by the simulation results. Our results open the avenue for an innovative means of specific and rapid detection of unknown bacteria for food safety and medicine from a nanoscale viewpoint.

## Introduction

Molecular recognition proposed by C. J. Pedersen, D. J. Cram, and J. M. Lehn governs the selective binding of biomolecules and crucial chemical reactions that occur in vital functions of living organisms^1,2^, including the formation of double-stranded DNA^3^, antigen-antibody reaction^4^, and sugar-lectin interactions^5^. A certain type of polymer molecule has an ability to transcribe the three-dimensional structure of a target molecule into a film produced by electropolymerization, namely, the molecular imprinting process (MIP)^6,7^, which has been utilized for the specific detection of amino acid enantiomers, metallic ions, and adenosine triphosphate (ATP)^8–10^. Although MIP has been limited to small molecules, a recent development in MIP has enabled the immobilization of macroscopic bacteria of micrometer dimensions^11^. Several previous studies have focused on the detection of bacteria, with regard to the selectivity of these molecular recognition processes^12^. For example, the antigen-antibody reaction utilized in immunoassays such as the enzyme-linked immunosorbent assay^13,14^ has been explored for bacterial detection^15,16^, where only a particular type of antibody can be used depending on the bacterial species. In addition, the identification and synthesis of a new antibody exhibiting specific binding with the target bacterium require an excessive amount time and effort. The detection of bacteria based on specific binding of their surface to a monosaccharide, mannose has been reported^17^, where a quartz crystal microbalance (QCM) with a mannose-modified electrode was used for the detection of mass change; however, the number of detectable bacterial species is limited. With regard to the MIP, it has been clarified that a specific bacterium (*Pseudomonas aeruginosa*) can be incorporated into the polypyrrole (PPy) film via electropolymerization and that by removing the cells during the over-oxidation process, several cavities are created in the over-oxidized polypyrrole (OPPy) film^18,19^. These cavities enable the highly selective trapping of the incorporated bacteria by guiding them to the film surface via dielectrophoresis. Bacterial surface antigens differ among serotypes^20,21^ and previous studies implied that the surface chemical structure (SCS) of incorporated bacteria was imprinted into these cavities; however, the physicochemical mechanism of this specific capture remains unsolved. Moreover, the development of BIF for multiple kinds of bacteria under a mixed condition was necessary for the real sample inspection, but it has been a challenging subject.

Regarding methods to guide bacteria toward the observation area, several papers using electric and light fields to enhance both the detection sensitivity of multiple bacteria and the efficiency of obtaining bacterial counts have been reported^22^. For example, highly sensitive detection (<1000 cells/mL) methods were proposed in passive manners with an electrochemical detection with bacteriophage^23^ and a piezoelectric mechanical detection with mannose^17^, whereas the selectivity was limited to a certain kind of bacteria depending on the probes and the detection time is longer than several tens minutes. Other group reported the capacitive biosensors with MIP provided the low detection limit less than 100 CFU/mL, whereas the time for the detection is on the order of several tens minutes^24^. Also, thermal wave transport analysis mediated by MIP exhibited high selectivity for several different bacteria, whereas the detection limit was larger than 10,000 cells/mL^25^. Various approaches have been explored, including dielectrophoresis using an alternating electric field between electrodes for actively guiding small and electrically-neutral objects toward the detection part^26,27^, optical tweezers using light-induced force based on electromagnetic interaction for contact-less trapping of a few objects^28,29^, and photothermal assembling using light-induced convection for the assembly of a large number of objects^30–32^. A unified theoretical framework of the driving force for the manipulation of electrically neutral materials by arbitrary oscillating electromagnetic fields can be described with the Lorentz force equation^33^. Additionally, based on this framework, theoretical methods for movement of small objects using oscillating electromagnetic fields with thermal fluctuation at room temperature have been developed for simulations in the time and energy domains, which enable evaluation of the dynamics involved and exploration of stable configurations^34,35^. These methods successfully explain the assembly process of metallic nanorods under laser light irradiation to obtain desired properties^36^. On the other hand, the mechanism of a dielectrophoretic manipulation of bacteria is also a crucial subject to design the substrate for selective trapping of target bacteria based on MIP.

Here, we created a BIF using the mixed liquid of different well-known bacteria as targets by transcribing their SCS into the OPPy film on the QCM disk electrode based on MIP, and our developed mixed BIF for multiple kinds of coliform group bacteria in food can be used for the detection of target bacteria in the real samples. Furthermore, we investigated the mechanism of selective bacterial trapping by altering the surface and internal conditions damaged with physicochemical manners. Particularly, we systematically investigated the principle underlying selective trapping in the BIF with an alternating electric field based on a dynamic Monte Carlo simulation with a stochastic approach in energy region taking into account the molecular recognition process and electromagnetic interaction. In addition, we created BIFs containing information regarding the surface structure of untreated, living bacteria and investigated the QCM frequency changes with dielectrophoresis using liquid samples containing bacteria damaged by thermal treatment, ultraviolet (UV) irradiation, and antibiotics. Subsequently, we demonstrated the effect of changes in bacterial conditions on specific capturing of bacteria and the feasibility of bacterial serotype analysis.

## Results

### Specific detection of bacteria using bacterial imprinted film and dielectrophoresis

**Figure 1:**
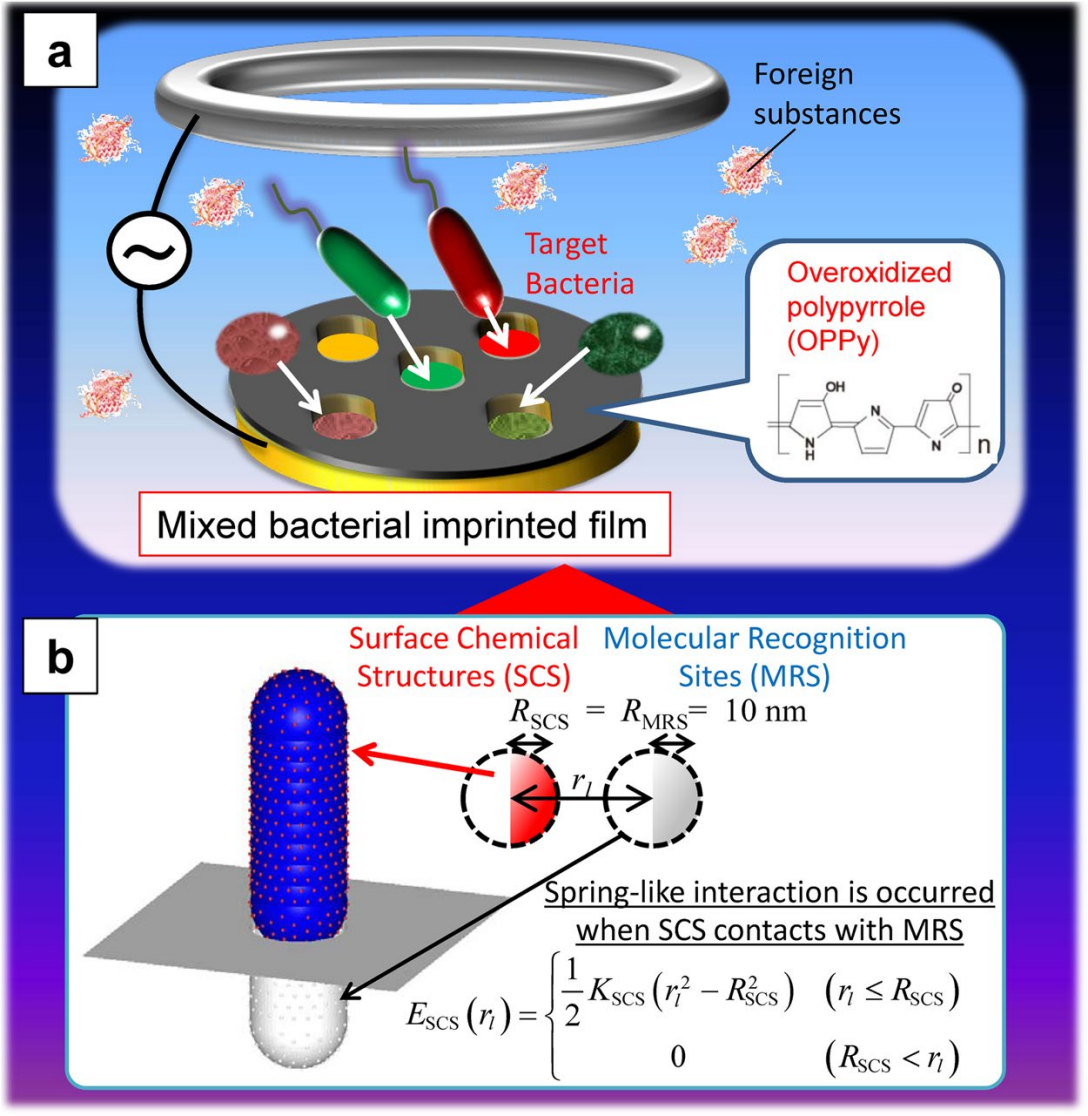
Concept of specific bacterial detection with mixed bacterial imprinted film. (**a**) Schematic illustration of specific detection of target bacteria with mixed bacterial imprinted film consisting of overoxidized polypyrrole (OPPy) and (**b**) model for the theoretical analysis. In (**a**), the meaning of colors (red, green, brown) of bacteria indicates the difference of their surface chemical structures (SCSs).

**Figure 2:**
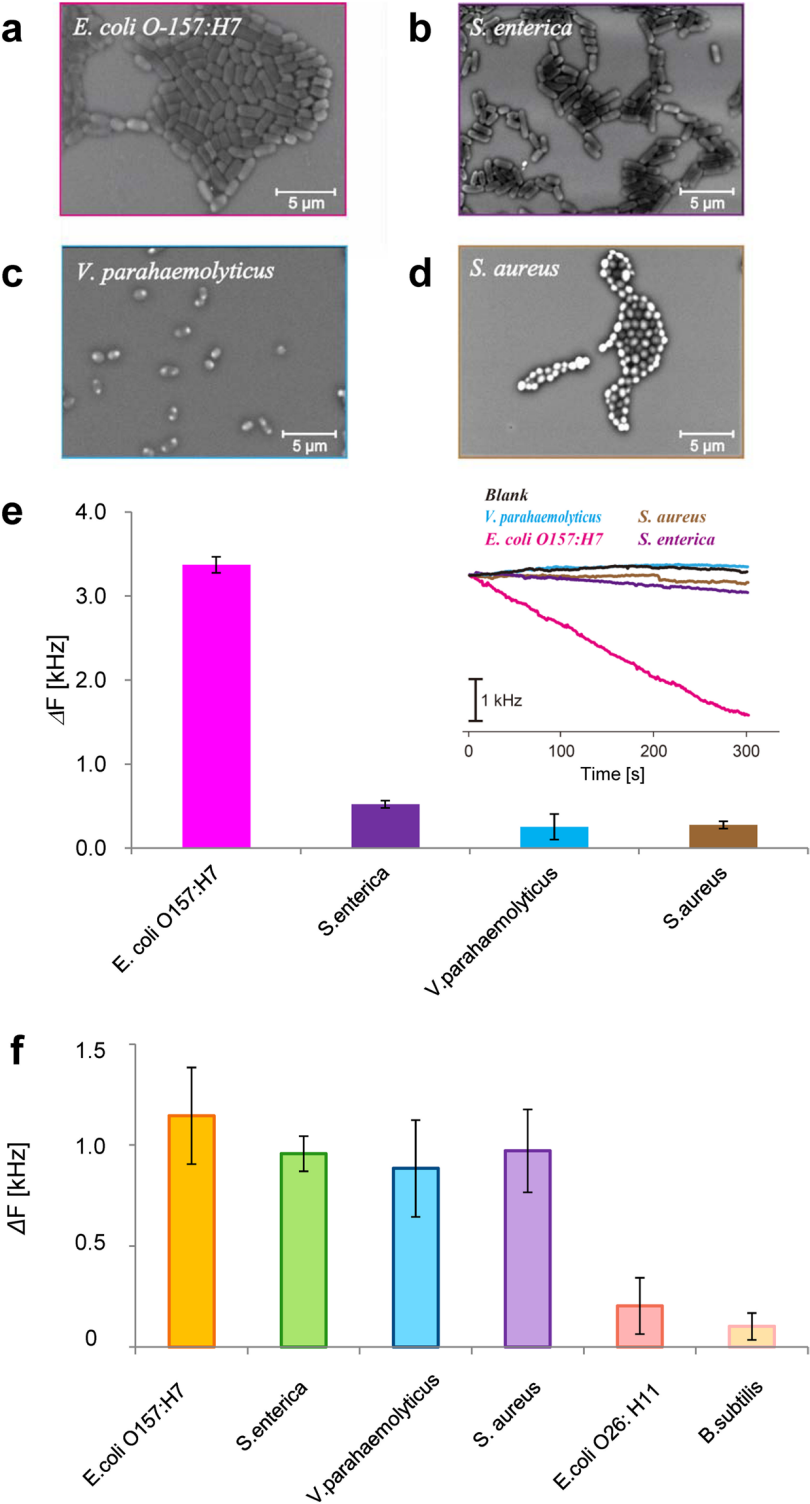
Specific detection in pure samples and demonstration of mixed bacterial imprinted film. (**a**)–(**d**) Scanning electron microscope (SEM) images of 4 types of bacteria. (**e**) The results of specific detection of *E. coli O157:H7* with the imprinted film of *E. coli O157:H7* in pure samples. Detection time was 300 s. Inset; the time dependence of QCM frequency for 4 types of bacteria including *E. coli. O-157:H7* (pink line). Black line indicates the result with no bacteria, and other lines indicate the results for *S. enterica*, *V. parahaemolyticus*, and *S. aureus*. The concentration of bacteria was 1 × 10^8^ CFU/mL. (**f**) Specific detection of complementary 4 types of bacteria with mixed bacterial imprinted film in pure samples. 2 types of mismatched bacteria (*E. coli. O26: H11*, *B. subtilis*) were also dispersed in the sample droplet. Detection time was 300 s. In Fig. (**e**), (**f**), the average of three time measurements was taken for each bacterium. Vertical bars indicate the standard deviation of the frequency change.

Figure 1 shows a schematic illustration of the device used in this study. Target bacteria were specifically detected by measuring changes in the mass of the OPPy film at the gold electrode using QCM, where the OPPy film imprinted with the target information was formed after incorporating bacteria during the preparation of PPy via electropolymerization and removal of bacteria via bacteriolysis (further details are provided in the Methods section). *Escherichia coli O157:H7* (*E. coli O157:H7*) was used for the formation of BIF, and 4 types of bacteria as follows, *E. coli O157:H7*, *Salmonella enterica* (*S. enterica*), *Vibrio parahaemolyticus* (*V. parahaemolyticus*), *Staphylococcus aureus* (*S. aureus*) shown in the scanning electron microscope (SEM) images in Fig. 2a–d, were detected in this study. As shown in Fig. 2e, liquid containing each bacterial species was individually dropped onto the BIF surface and changes in QCM frequency were measured after dielectrophoresis (frequency: 10 MHz, voltage: 20 Vpp). Frequency changes for the droplet containing *E. coli O157:H7* exhibited an order of magnitude greater than those for the other non-target bacteria. The shape and size of *E. coli O157:H7* and *V. parahaemolyticus* as bacillus (rod-shaped bacterium) were almost the same, whereas the shape and size of *V. parahaemolyticus* and *S. aureus* as coccus (spherical bacterium) were almost the same. This result and SEM images implied that SCS of these bacteria were transcribed into the OPPy film and lead to the difference of frequency change even for the same shape and size.

In particular, electropolymerization and OPPy film preparation were performed using a liquid containing a mixture of the 4 target bacterial species as shown in Fig. 2a–d, i.e., a mixed BIF was produced after removing all bacteria. The results of frequency change measurements during dielectrophoresis are shown in Fig. 2f, where 6 types of bacteria including the targets were individually dropped onto the mixed BIF. Remarkably, samples containing the above-mentioned 4 target bacterial species exhibited clear frequency changes, with successful specific capture even with the mixed BIF. Furthermore, there was almost no frequency change with the samples containing 2 types of non-target bacteria (*E. coli O26:H11* and *Bacillus subtilis*). These results indicate the high selectivity and specific detection ability of the mixed BIF. Also, the measurements in Fig. 2f were performed using the same mixed BIF, and the high reproducibility was confirmed even after the several tens times measurements.

### Detection of damaged bacteria

**Figure 3:**
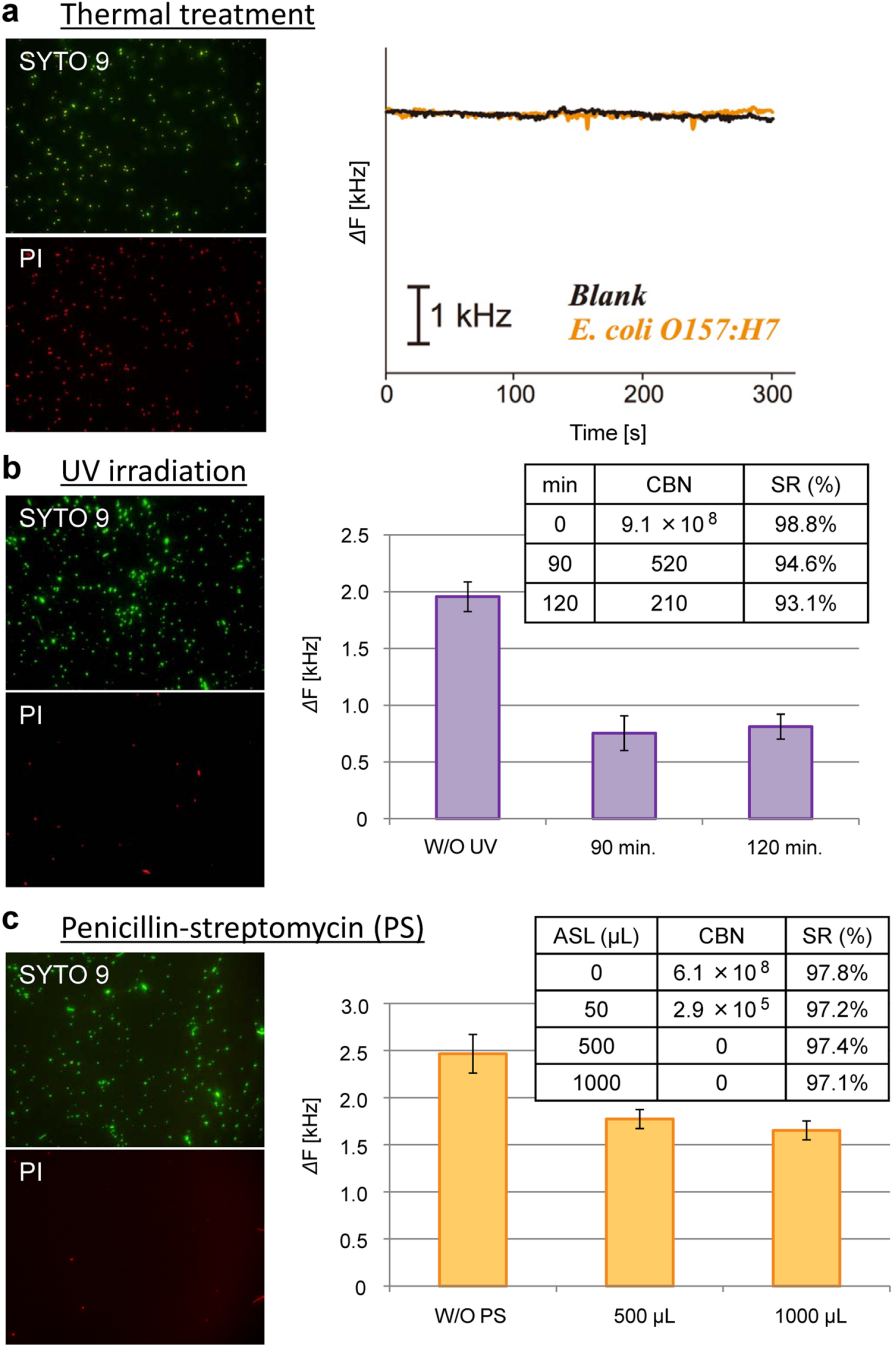
Specific detection of outage bacteria (*E. coli O157:H7*) damaged by different methods. (**a**) thermal treatment, (**b**) ultraviolet (UV) light irradiation, and (**c**) antibacterial agent (penicillin-streptomycin; PS). W/O means “without”. Cultivated bacteria number (CBN) and Surface-maintained rate (SR) are summarized in the inset tables of (**b**) for different UV irradiation times, and of (**c**) for different amounts of sample liquid (ASL). In Fig. (**b**), (**c**), the average of three time measurements was taken for each column graph. Vertical bars indicate the standard deviation of the frequency change.

In order to investigate the effect of surface structure on the specificity of BIF, QCM frequency changes after dielectrophoresis were measured after damaging the bacteria via thermal treatment, UV irradiation, and an antibiotic agent (Fig. 3, The detailed information on each treatment is shown in Methods section). There were almost no frequency changes in the case of thermal treatment (Fig. 3a), while the frequency was reduced to 1/3 of the baseline value in the case of UV irradiation for >90 minutes (Fig. 3b). Although the bacterial cell was expected to be destroyed or damaged by the antibiotic agent, a high QCM frequency change was maintained even with high concentrations of the antibiotic, namely 1000 μL penicillin-streptomycin (Fig. 3c).

### Theoretical analysis of specific detection of bacteria

**Figure 4:**
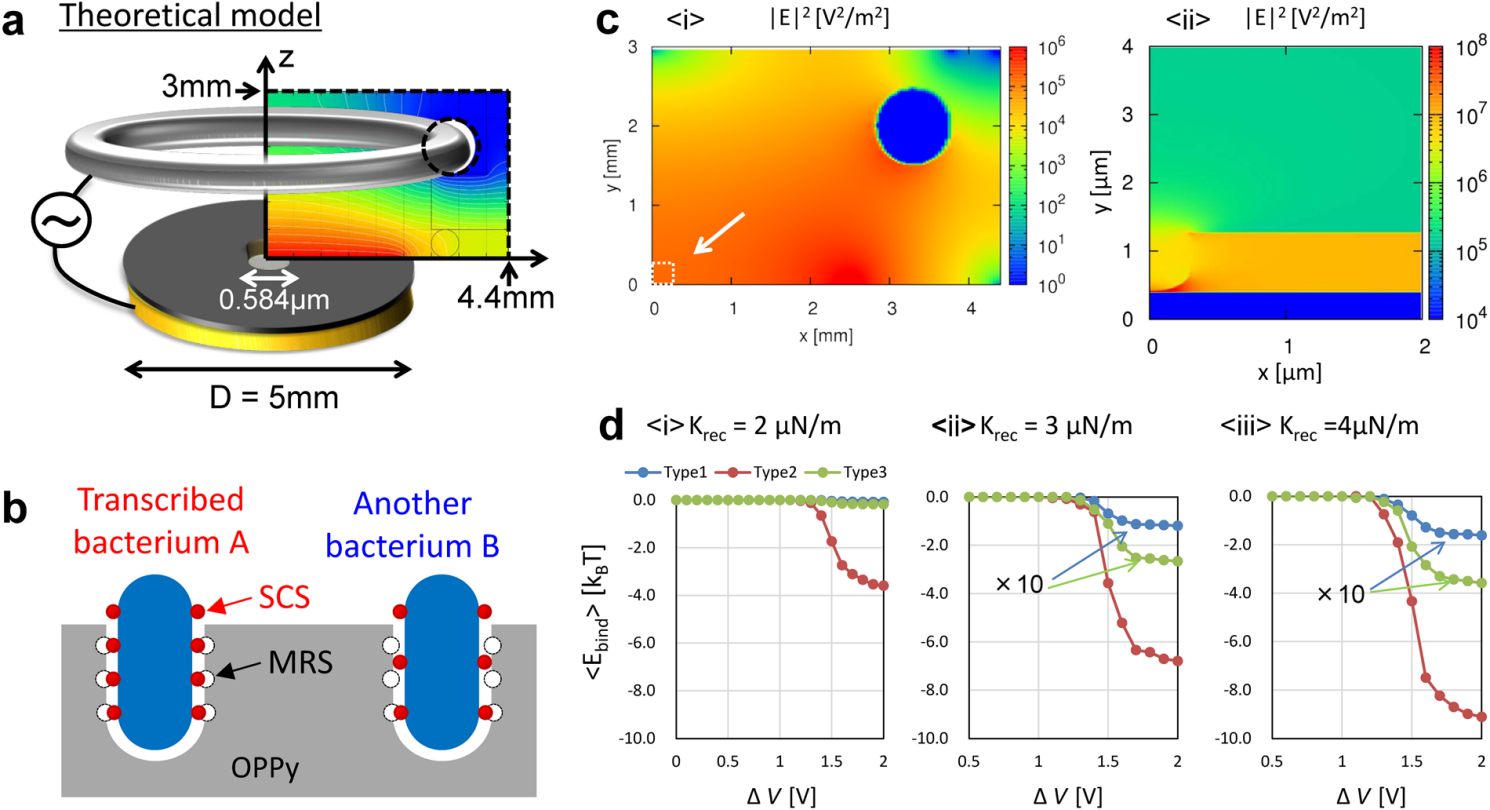
Schematic images of (**a**) electrodes and electric field, and of (**b**) bacterial imprinting with target bacterium (Complementary bacterium A and mismatched bacterium B. Surface chemical structures (SCS) were assumed to be present on each bacterium, and molecular recognition sites (MRS) were assumed to be present in the OPPy film. SCS were modelled with red spheres of 10 nm diameter, and MRS were assumed to be in a hole (bacterial cavity). (**c**) 〈i〉 Spatial distribution of electric field around ring electrode and disk electrode, and 〈ii〉 the enlarged figure near the center of disk electrode (enclosed with white dotted square). The electrical potential difference between the center of disk electrode and the ring electrode (Δ*V*) was set to 1 V, where the electric field is proportional to Δ*V* and maintain the spatial distribution. (**d**) 〈i〉–〈iii〉 Numerically averaged total binding energy during 0.5 × 10^5^–1.0 × 10^5^ steps in dynamic Monte Carlo simulation was plotted as a function of Δ*V* for each value of *K*_rec_. Sample average was also evaluated after 100 trials.

Figure 4 details the Molecular Recognition Metropolis Method (MRMM; details are provided in the Methods section) used in this study, for the theoretical analysis of the mechanism of high selectivity of BIF. The distribution of applied alternating electric field between the ring electrode and the OPPy-coated disk electrode for the dielectrophoresis was calculated using the finite difference method (FDM) as described in Fig. 4a. The SCS on each bacterium was modeled using a nanoscale sphere and a corresponding imprinted molecular recognition site (MRS) was modeled with a nanoscale dip whose size is almost the same as the SCS, as shown in Fig. 1 and Fig. 4b. Under these assumptions, light-induced molecular recognition method^32^ was optimized for configuration control of a bacterium with low frequency electric field, i.e., MRMM as the method for the generalized electromagnetic field. For the essential discussion, it was assumed that no interaction occurred between bacteria and a single cavity was present at the center of OPPy film after removing a bacterium (bacterial cavity).

Next, the spatial distribution of applied electric field was plotted in and around the bacterial cavity after removing a single bacterium at the center of OPPy film on the disk electrode (Fig. 4c). The energy of random motion of a bacterium was assumed to be 1 *k*_B_ *T* (*k*_B_ is the Boltzmann constant, and *T* = 298.15 K near the value at room temperature). Figure 4c 〈i〉 indicates that electric field was high over a wide region between the ring electrode and disk electrode. In the enlarged panel within Fig. 4c 〈ii〉, the electric field at the bottom of the bacterial cavity was greatly enhanced, to approximately 100 times the value around the cavity. Clausius-Mossotti factor related with polarizability of a bacterium (*E. coli* was assumed) was positive as evaluated for the experimentally used frequency of 10 MHz (Supplementary Figure S1), and the bacterium was expected to be attracted into the bottom of the cavity (See Methods section and Supplementary Figure S2). Additionally, as shown in Fig. 4d 〈i〉–〈iii〉, the averaged value of binding energy between a bacterium and a bacterial cavity (〈*E*_bind_〉) during 0.5 × 10^5^ – 1.0 × 10^5^ steps in the Monte Carlo simulations was plotted as the function of the voltage amplitude as the electrical potential difference between the disk electrode and the ring electrode (Δ*V*) by changing the spring constant (*K*_rec_) as the indicator of the binding strength between a pair of SCS and MRS for 3 types of bacteria. The frequency change of QCM (*ΔF*) has a finite value only when *K*_rec_ is not zero, and this means that *ΔF* depends on *K*_rec_ according to the model calculation of coupled harmonic oscillator. Type 2 corresponds to the “*transcribed bacterium A*” in Fig. 4b, which exhibits high complementary with the cavity, since the spatial configurations of SCSs were perfectly matched with the configurations of MRSs. On the other hand, each spatial configuration of SCSs in Type 1 or Type 2 was different from the configurations of MRSs in the cavity (“*another bacterium B*”) as shown in the spatial configuration of SCS in Supplementary Figure S3. The total sum of binding energy became −3.0 *k*_B_ *T* for *K*_rec_ = 1 μN/m if all the SCSs of the lower part of bacterium were specifically bound to all the MRSs (250 pairs), which is lower than 〈*E*_bind_〉. The binding energy of each pair of SCS and MRS was proportional to *K*_rec_, and the probability of the selective trapping increased for large *K*_rec_. It was clarified that the common feature in each value of *K*_rec_ was that 〈*E*_bind_〉 for complementary bacterium (Type 2) achieves an order of magnitude greater than those for mismatched bacteria (Type 1 and Type 3), which corresponds well with the experimental results shown in Figs 2–3.

### Specific detection with real samples

**Figure 5:**
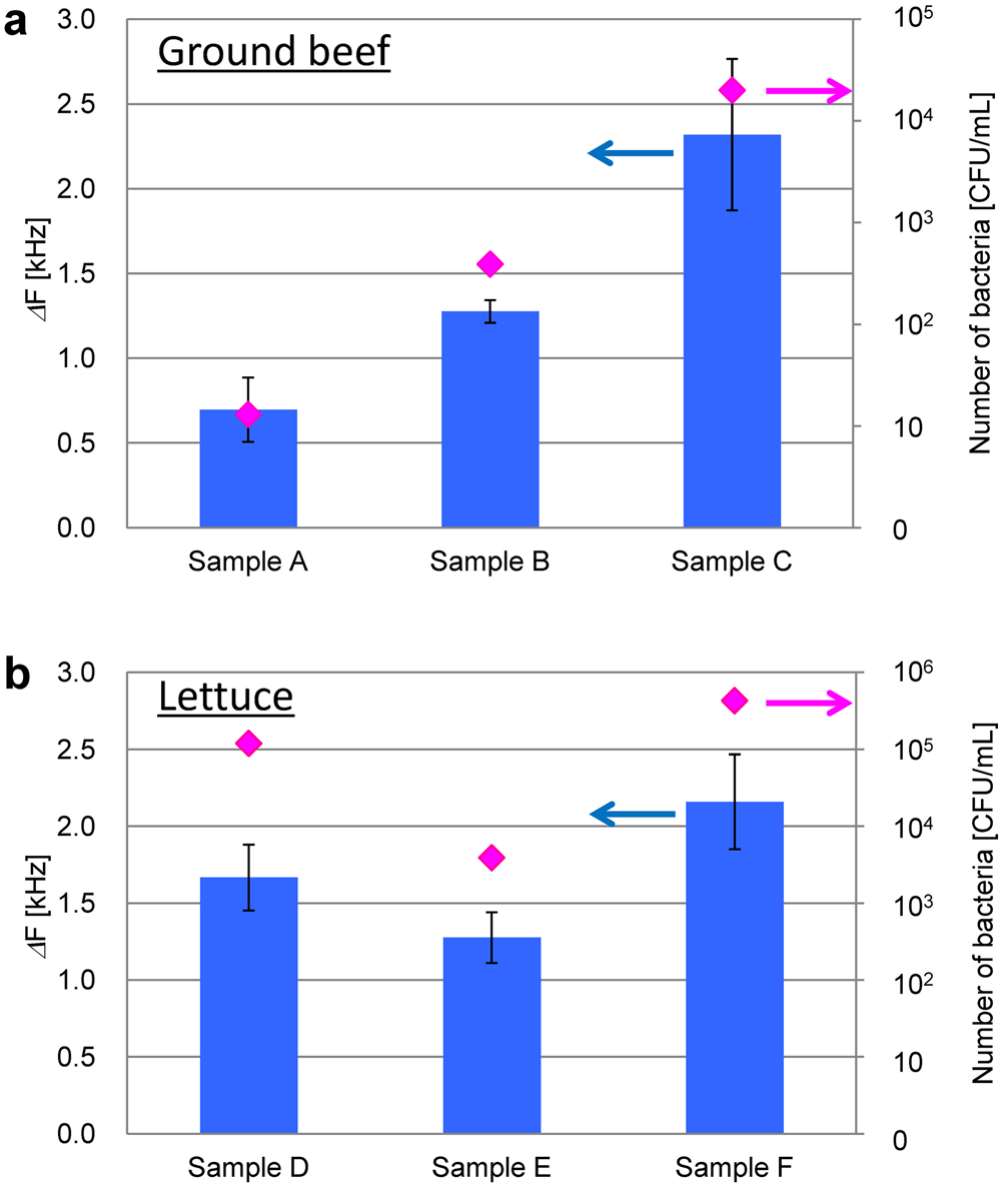
Specific detection of bacteria in real samples. (**a**) Filtered liquid of stomached ground beef. (**b**) Filtered liquid of stomached lettuce. In Fig. (**a**), (**b**), the average of three time measurements was taken for each column graph. Vertical bars indicate the standard deviation of the frequency change.

Figure 5 shows the results of bacterial detection in ground beef and lettuce as real food samples (the experimental detail is in Methods section). In Fig. 5a, 25 g of ground beef was stomached (230 rpm, 30 s) in 225 mL phosphate buffer, and the extracted liquid was filtered through a gauze to remove lipid to obtain the real sample. In Fig. 5b, 3 lettuce leaves were stomached under similar conditions and filtered to prepare the real sample. Preliminarily, bacteria in each sample were cultured on the 3 M Petrifilm for the coliform group, and bacterial counts were estimated. The frequency change in QCM was confirmed as 10 colony-forming units (CFU)/mL in the case of ground beef, and 10^3^ CFU/mL in the case of lettuce. The samples A–F were purchased at different supermarkets. The bacterial count in Sample C was the highest, followed by Samples B and A. The frequency changes in QCM with BIF also showed the same order and a high correlation was confirmed between the culture method and our method. Moreover, the order of the bacterial counts estimated from the frequency change was F, D, E, which indicates a high correlation between both methods and high reliability of our developed detection method using BIF.

## Discussion

First, we discuss the results of bacterial detection in pure samples without foreign substances using alternating electric field and individual BIFs (Fig. 2) and the mixed BIF (Fig. 2f). In Fig. 2e, a clear frequency change was confirmed using QCM with BIF transcribed with the information of *E. coli O157:H7* by adding *E. coli O157:H7*, whereas almost no frequency change was noted after adding other bacteria (*S. enterica*, *V. parahaemolyticus*, *S. aureus*) as shown in Fig. 2a–d. It should be stressed that a clear difference in frequency change was also observed within a very short time (300 s) not only between the bacillus (rod-shaped bacterium) and the coccus (spherical bacterium), but also between bacteria of similar size and shape (*E. coli O157:H7* and *S. enterica*). This result implies that the information of SCS as modeled in Figs 1 and 4b was accurately transcribed as MRS into the OPPy film. Thus, our method can be used for arbitrary strain types with high selectivity since the visible change more than 1 kHz was observed even within 1 minute as shown in the inset of Fig. 2e. Furthermore, even in the real samples, there is a possibility that the present lowest detection limit was 10^2^ CFU/mL of bacteria can be detected from Fig. 5a, where the detection range was 10^2^–10^6^ CFU/mL. These values mean that our method has advantages from multiple viewpoints of selectivity, response time, sensitivity, detection limit, types of strains, in comparison with Refs.^17,23–25^. If the light-induced assembling in Ref.^31,32^ would be used for the guiding process of bacteria into BIF, the remote condensation of bacteria without counter electrode would be possible, which is a future subject.

As shown in Fig. 2f, we created a mixed BIF transcribed with 4 types of bacteria (*E. coli O157:H7, S. enterica*, *V. parahaemolyticus*, *S. aureus*: Fig. 2a–d), and individual detections were performed for 6 types of bacteria including *E. coli O26:H11* and *B. subtilis* that were not used in the production of the BIF. Clear frequency changes were observed when adding the 4 target species but the frequency changes for the latter 2 types of bacteria were negligible, which indicates that *E. coli O26:H11* and *B. subtilis* were not trapped by the mixed BIF. These results strongly support specific detection of target bacteria by guiding them toward the BIF even in the real samples containing various types of bacteria, and BIF created with various groups of bacteria exhibited high selectivity.

In order to clarify the mechanism of the selectivity, we explored the availability of specific detection of *E. coli O157:H7* damaged by 3 methods (Fig. 3). The 1st method was thermal treatment^37^, and propidium iodide (PI) staining shows red fluorescence. This means that the cell membrane was damaged by heat and PI entered the bacterial cell (Fig. 3a) since SCS were broken. In addition, there was no frequency change in QCM and no bacteria were trapped. The 2nd method was UV irradiation, and PI did not enter the bacterial cell as the membrane was not damaged; however, the internal functions of the bacterium were inhibited and the sugar chains on the cell surface were damaged^38^, which can be explained by the decrease of CBN in the inset table of Fig. 3b. Therefore, the QCM frequency change decreased after a long period of UV irradiation and the bacterial count failed to increase even after bacterial cultivation (Fig. 3b). The 3rd method included the use of an antibiotic that penetrated the cell membrane without causing damage^39^. In this case, the bacterial count did not increase due to high concentration of the drug. This indicates that the bacteria were either killed or functionally inactive, but the QCM frequency change maintained a high magnitude, at 70% even when the amount of the drug was increased (Fig. 3c). This result indicates that the specific binding was not affected by antibiotic treatment. From these facts, we can understand that MRS in BIF consisting of OPPy sensitively recognizes the SCS of the original bacteria.

Moreover, we performed the theoretical calculations based on dynamic Monte Carlo method to understand the mechanism of high selectivity considering the bacterium Type 2 with the configurations of SCSs complementary to MRSs and mismatched bacteria Type 1 and Type 3 with different SCS configurations as shown in Fig. 4. All types of bacteria were once guided into the bacterial cavity by the application of electric field (Fig. 4d), Supplementary Movie 1–3, the electric field was set to Δ*V* = 1.5 V and turned off at the 1 × 10^5^ step during 2 × 10^5^ step). However, in spite of fluctuating Δ*V* value between 0–2 V, the averaged binding energy < *E*_bind_ > for Type 2 was always highest, at −4.3 *k*_B_ *T* for *K*_rec_ = 4 μN/m and Δ*V* = 1.5 V. In this case, trapping for a long period was achieved even after turning off the electric field as shown in Supplementary Movie 2 (if all the SCSs were bound to MRS, the total binding energy can be −12.1 *k*_B_ *T*). Meanwhile, Type 2 bacterium was trapped during certain steps after turning off the electric field, but finally escaped from the cavity, where the <*E*_bind_>  = −3.6 *k*_B_ *T* for *K*_rec_ = 3 μN/m and Δ*V* = 1.5 V (if all the SCSs were bound to MRS, the total binding energy can be −9.1 *k*_B_ *T*). These results indicate stable specific binding, where the maximum magnitude of binding energy was on the order 10 *k*_B_ *T*, which is the assumed energy of random motion of a bacterium. This indicates that the enhancement of the attractive interaction leads to a coupled oscillator of the QCM electrode and only the bacteria specifically trapped in the cavities for the complementary configuration of SCSs and MRSs, where the strong coupling gives a prominent frequency change via mass addition. On the other hand, we have confirmed that the dielectrohpretic force (the gradient of electromagnetic potential by the alternating electric field) may play a crucial role since there was no frequency change in QCM under the guiding of bacteria toward the surface of BIF by a pressure-driven flow without electric field. The results strongly suggest the probability of specific trapping of bacteria was increased due to molecular recognition between target bacteria and corresponding cavities, thus allowing identification of bacteria with similar shape and size. This tendency in Fig. 4c and d corresponds well with the large frequency change for the detection of normal *E. coli O157:H7* with a single BIF for *E. coli O157:H7* in Fig. 2e. Furthermore, if the SCS would be damaged by UV irradiation or heat treatment, *K*_rec_ would get smaller and decrease the difference between the averaged total binding energy <*E*_bind_> for the complementary bacterium and <*E*_bind_> for the mismatched bacterium. Therefore, even in case that the spatial configuration of each SCS is the same, it is considered that the selectivity of BIF would change after each SCS was damaged as shown in the experimental results in Fig. 3a and b. On the other hand, as discussed in the previous paragraph, the QCM frequency change maintained 70% magnitude even in the case of *E. coli O157:H7* damaged by antibiotic drug treatment in Fig. 3c since there is almost no surface damage. When the surface of each bacterium was damaged by thermal treatment and UV irradiation, the frequency change dramatically decreased. Thus, the specific binding of SCS on the surface of bacteria and MRS in the bacterial cavity in the nanoscale region leads to the macroscopic selective trapping.

Especially, even in the experiments with real samples extracted from meat and vegetable (Fig. 5), we also confirmed rapid specific detection of coliform group and a high correlation with the standard culture method (coliform group is generally regarded as an indicator of contamination). Meat contains higher lipid content compared with vegetables and the bacterial concentration in ground meat was estimated to be one order lower via the QCM frequency change compared with that of vegetable (Fig. 5a and b). Although an additional pretreatment was performed to remove lipids from ground meat, specific binding processes were blocked due to the remained lipids on the BIF. However, the frequency changes depending on the samples A, B, C indicated specific binding of SCS and MRS, and there was a certain level of correlation with the results of culture method. This is an important fact that we can specifically detect bacteria even under lipid-rich conditions. These results correspond well to the results shown in Fig. 2f, and indicate high feasibility of using mixed BIF to detect pathogens causing food poisoning.

In summary, we have successfully produced a mixed bacterial imprinted film and demonstrated a clear difference in selectivity between active and surface-damaged bacteria. Furthermore, an important fact regarding the origin of the high selectivity was obtained from the theoretical analysis based on MRMM, optimized for the bacteria and low frequency electric field taking into account the molecular recognition sites with coarse grained model. Especially, we revealed that our developed bacterial imprinted film can analyze the serotype of bacteria, and can be used for the cyclopedic detection of the coliform group even in lipid-rich foods. In the future, the potential applications of developed imprinted film can be explored and the binding process of surface chemical structure and molecular recognition site can be evaluated using a quantum chemical calculation. This will further clarify the mechanisms of high selectivity. Successive evolution of the bacterial imprinted film will create a novel mechanism of bacterial detection in food safety as well as more diverse medical practices.

## Methods

### Chemicals and Materials

All chemical reagents were of analytical grade, used as supplied without further purification unless indicated. Pyrrole, KH_2_PO_4_, H_3_PO4, lysozyme, sodium dodecyl sulfate (SDS), and NaOH were purchased from Wako Pure Chemical Industries (Japan). Nutrient broth was purchased from Eiken Chemical (Japan). Bacterial viability kit L7007 (SYTO9/PI) was purchased from Molecular Probes. Bacteria such as *E. coli O157:H7* and *E. coli O26:H11* were provided by Prof. Miyake in Osaka Prefecture University. On the other hand, *Salmonella enterica*, *Vibrio parahaemolyticus*, *Staphylococcus aureus, and Bacillus subtilis* were purchased from the Biological Resource Center (NBRC), National Institute of Technology and Evaluation, Japan. Milli-Q grade (>18 MΩ) water with ultraviolet sterilization was used throughout the study.

### Preparation of OPPy Film with Imprinted Bacterial Configuration (pure samples)

An AT-cut gold-evaporated quartz crystal microbalance (QCM) electrode (Seiko EG&G; surface area, 0.2 cm^2^) was pretreated by soft plasma etching (Meiwa Fosis, SEDE-GE) for 30 s. A PPy films doped with the bacteria (*E. coli O157:H7*, *S. enterica*, *V. parahaemolyticus*, *S. aureus*) were individually prepared on the QCM electrode by applying a constant potential of +0.98 V vs Ag/AgCl/sat. KCl, in a 0.2 M phosphate buffer solution (pH 2.56) containing 0.1 M pyrrole and bacteria of the total concentration 1.0 × 10^8^ CFU/mL (the concentration of each kind of bacterium was 0.25 × 10^8^ CFU/mL for the mixed BIF) with a potentiostat (model 842B, ALS). A thin black film was formed on the electrode after electrochemical polymerization for 90 s.

As the next step, overoxidation treatment was applied to create the shape-complementary cavities in the PPy film. Before the overoxidation, we pretreated the film with lysozyme (30 mg/mL) and 5% SDS for 48 hours at 30 °C to remove the strong interactions between polysaccharide on the cell wall and polymer surface. Subsequently, a constant potential (+0.98 V vs Ag/AgCl/sat. KCl) was applied in an aqueous sodium hydroxide solution (0.1 M) to overoxidize PPy with the bacteria used in this paper and to expel the negatively charged bacteria from the polymer texture^18,19^. The surface of the film was observed before and after overoxidation by a scanning electron microscope (SEM) with a TM-1000 miniscope (Hitachi, Japan).

### Bacterial Cultivation

Bacteria used in this study (30 °C for *E. coli O157:H7*, *S. enterica, E. coli O-26:H11, B. subtilis*, and 37 °C for *V. parahaemolyticus, S. aureus*) were cultured in 30 mL nutrient broth overnight around 30 °C^18^. The cultured bacteria were centrifuged at 6000 rpm for 10 min, and the precipitate was re-dispersed in 30 mL sterile water. This procedure was repeated at least 3 times to obtain purified target analyte (bacterial concentration, 10^8^ CFU/mL).

### Bacterial Detection Using OPPy Film

After addition of 500 µL sample bacterial suspension in sterilized water, the resonance frequency change of the OPPy modified electrode, having bacterial cavities, was monitored with QCM (model QCM934, Seiko EG&G), while the electric field (frequency, 10 MHz; voltage, 20 Vpp) was impressed between the QCM electrode and Pt counter electrode by using a waveform generator (7075, HIOKI, Japan) coupled with a power amplifier (HSA 4101, NF corporation, Japan)^18^. A schematic illustration of the electrode arrangement is shown in Fig. 1a. The Pt counter electrode was set up 1.5 mm above the QCM electrode.

### Fluorescence Microscope Observation of Viability and Surface Condition of Bacteria

In Fig. 3, in order to confirm the condition of bacteria (*E. coli O157:H7*), they were stained by a fluorescent pigment such as SYTO9/PI, which is often used for bacterial viability assay. The fluorochrome assay stains the nucleic acid of bacteria: PI stains the dead cells red by penetrating through the damaged cell membranes, while SYTO 9 fluorescent stain labels all the cells green. The fluorochrome reagent was used, following the manufacturer instructions. The conditions of damaged bacteria after the thermal treatment (80 °C, 20 min.), UV irradiation (TOSHIBA, GL15; wavelength is 253.7 nm, and intensity is 51 μW/cm^2^) and antibiotic drug treatment (penicillin-streptomycin (PS) solution purchased from WAKO; 10,000 units/mL penicillin G, 0.85% saline solution containing 10,000 μg/mL streptomycin sulfate) were observed under a fluorescent microscope (BX51, Olympus), focusing on the OPPy film formed on the Au ring-disk electrode (NTT-AT; surface area, 0.07 cm^2^). In the case of antibiotic drug treatment, PS solution with each amount (50 μL, 500 μL, 1000 μL) was added to 5 mL of dispersion liquid of bacteria, and incubated at 37 °C for 1 hour. In all the three methods, 1 mL of treated liquid was used for each detection after washing with sterile water. The fluorochromes were excited under illumination of 460–490 nm and observed through high-pass optical filters with a cut-off wavelength of 520 nm. Cultivated bacterial numbers (CBN) were evaluated with 3 M Petrifilm after 12 hours. Surface-maintained rates (SR) were evaluated with the following equation; SR = (*N*_green_ − *N*_red_)/*N*_green_, where *N*_green_ is the number of green bright dots (SYTO 9) and is the number of red bright dots (PI).

### Preparation of bacteria imprinted film with coliform group in ground beef and lettuce (real samples)

Bacteria in commercially available ground beef (25 g) and 3 leaves of lettuce were used to create BIFs for the real samples, respectively. Preliminarily, bacteria in the ground beef were cultured for 6 hours at 30 °C in the thermostatic chamber (the bacterial number increases from 1.4 × 10^3^ CFU/mL to 1.1 × 10^4^ CFU/mL). Each sample was immersed in 225 mL phosphate buffered dilution water (pH 7.2) with the sterilized bag for the stomaching. After stomaching each sample (230 rpm, 30 s), the obtained stock solution was moved into 30 mL nutrient broth via a platinum loop. Moreover, after the shaking culture of bacteria in each sample was performed for 18 hours at 30 °C in the thermostatic chamber, 1.5 mL culture liquid was dispensed into microtubes and washed by sterile water. After this procedure, each bacterial sample was dispersed into 500 µL phosphate buffer containing PPy, and a constant potential of 975 mV was applied until frequency changed to 80 kHz, to create the PPy film. Removal of bacteria with over-oxidization and bacteriolytic reaction from the film was the same as mentioned above for pure samples.

### Bacterial Detection in real samples (ground beef and lettuce) using OPPy Film

We used the inspection method established by Japanese Ministry of Health, Labor and Welfare as follows. Commercially available ground beef (25 g) and three leaves of lettuce were individually immersed in 225 mL phosphate buffered dilution water (pH 7.2) and stomached in a sterilized bag (230 rpm, 30 s). Each sample was dispensed into a microtube (1000 µL) after stomaching, and the detection was performed (results shown in Fig. 5) by the same procedure as that used for the pure samples.

### Molecular recognition Metropolis Method for Specific Detection of Bacteria

The MMRM^32^ model for bacteria and bacterial imprinted film with low frequency electric field based on the stochastic method under the self-consistently determined electric field with Maxwell equations for arbitrary frequency is illustrated below. The model for specific binding of a bacterium and bacterial imprinted film is shown in Figs 1 and 4b (see also Supplemental Movies S1–S3). By solving the Maxwell equations based on a discretized integral with spherical cells (DISC)^34^, the response field E and induced polarization P were evaluated as follows:

1 $${{\bf{E}}}_{i}={{\bf{E}}}_{i}^{(0)}+\sum _{j\ne i}^{N}{{\bf{G}}}^{{\rm{hm}}}({{\bf{r}}}_{ij})\cdot {{\bf{P}}}_{j}{V}_{j}+{{\bf{S}}}_{i}\cdot {{\bf{P}}}_{i},$$

2 $${{\bf{P}}}_{j}={{\boldsymbol{\chi }}}_{j}\cdot {{\bf{E}}}_{j},$$

where each bacterium was modeled with *N* = 3 spherical cells (*N* is the number of cells), *N* is the number of cells, ${V}_{j}$ is the volume of each cell, ${{\bf{E}}}_{i}^{(0)}$ is the incident electric field, **G** ^hm^ is the non-retarded Green’s function in a homogeneous medium, and **χ**_*j*_ is the electric susceptibility tensor related with the dielectric function determined by the ellipsoidal core-shell model considering the longitudinal and transverse axis components for rod-like bacterium^40^ related with dielectric function of each cell. The integral ${{\bf{S}}}_{i}={\int }_{{V}_{i}}d{\bf{r}}{\boldsymbol{^{\prime} }}{{\bf{G}}}^{{\rm{hm}}}({{\bf{r}}}_{i}-{\bf{r}}{\boldsymbol{^{\prime} }})$ for *i* = *j* as the self-term in equation (1) was analytically calculated. By substituting the obtained **E** and **P** as solutions of the simultaneous equations (1) and (2) into the general expression of force by oscillating electromagnetic field.

3 $$ < {{\bf{F}}}_{i} > =(1/2)\mathrm{Re}\,[\sum _{\omega }{\int }_{{V}_{i}}d{\bf{r}}(\nabla {\bf{E}}{({\bf{r}},\omega )}^{\ast })\cdot {\bf{P}}({\bf{r}},\omega )]$$

We can also evaluate the dielectrophoretic force acting on bacteria floating in the medium without imaginary part of dielectric constant while this equation was derived for the evaluation of light-induced force by visible electromagnetic field. For the low frequency electric field in dielectrophoresis, the wave number can be *q = ω/c* ≈ 0 in **G** ^hm^ and **S**_*i*_, approximately.

The frequency dependence is contained in **χ**_*j*_, and the induced polarization of each spherical cell in a bacterium as a dielectric object can be given as

4 $${\bf{P}}({\bf{r}},\omega )=\sum _{i=1}^{N}3{\varepsilon }_{0}{\varepsilon }_{{\rm{med}}}{K}_{i}(\omega ){{\bf{E}}}_{{\rm{inc}}}({\bf{r}},\omega ),$$

where ${K}_{i}(\omega )$ is the Clausius-Mossotti factor for the *i*th cell evaluated by solving Eq. (1) and (2), and *ε*_0_ is the vacuum permittivity, *ε*_p_ is the dielectric function of each cell, *ε*_med_ is the dielectric constant of the medium (water is assumed here). The dielectrophoreitic force on the *i*th cell of a bacterium can be given as

5 $$ < {{\bf{F}}}_{i}^{({\rm{DEP}})} > =(3/4){V}_{i}{\varepsilon }_{0}{\varepsilon }_{med}\mathrm{Re}[{K}_{i}(\omega )]\nabla |{\bf{E}}({\bf{r}},\omega ){|}^{2},$$

which is similar to the expression in Ref.^41^. In addition, the dielectric function of each cell in bacterium was determined by comparing the value obtained with the ellipsoidal core-shell model consistent with experimental value of *E. coli* ^40^. The incident electric field ${{\bf{E}}}_{i}^{(0)}$ between a ring electrode and a disk electrode in Fig. 4c was evaluated by solving the Poisson equation with finite difference method (FDM)^42,43^ assuming the model as shown in Fig. 4a, where both the ring electrode and disk electrode were assumed to be perfect conductor (detailed information of the electromagnetic boundary conditions and environmental parameters are in Supplementary Figure S4). Thus, Neumann boundary condition was applied on the boundary of simulation region, where the gradient of potential is 0. Also, the Dirichlet boundary conditions were applied on the circumference of ring electrode 0 (V), and on the surface of disk electrode ΔV (V).

As shown in Fig. 4b, different kinds of bacteria are modelled by changing the number and the configuration of SCS, because the difference in the shape of bacteria can be distinguished by the shape of the cavity. For the simplicity, only one kind of SCS was assumed for each SCS sphere on the surface of a bacterium. Taking into account the interaction between SCS and MRS, stochastic behavior of bacteria was analyzed by Monte Carlo method with Metropolis algorithm. The interaction potential is given by the sum of the change of dielectrophoreitic potential with the spatial displacement ${\rm{\Delta }}{{\bf{r}}}_{i}$ and the total binding energy of *M* pairs of SCS and MRS ${E}^{({\rm{Bind}})}=\sum _{l=1}^{M}{E}_{l}^{({\rm{SCS}})}({r}_{l})$ as follows

6 $${\rm{\Delta }}H=-\sum _{i=1}^{N} < {{\bf{F}}}_{i}^{(\mathrm{DEP})} > \cdot {\rm{\Delta }}{{\bf{r}}}_{i}+{\rm{\Delta }}{E}^{({\rm{Bind}})},$$

where the binding energy between SCS and MRS in the the *l*th pair can be given by the spring model^44^ as follows

7 $${E}_{l}^{({\rm{SCS}})}(r)=\{\begin{array}{cc}\frac{1}{2}{K}_{{\rm{SCS}}}({r}_{l}^{2}-{R}_{{\rm{SCS}}}^{2}) & ({r}_{l}\le {R}_{{\rm{SCS}}})\\ 0 & ({r}_{l} > {R}_{{\rm{SCS}}})\end{array}$$

When the center distance between a SCS and a MRS (*r*) becomes less than the effective radius of SCS (${R}_{{\rm{SCS}}}$), specific binding can occur from Equation (7). In the simulation, we changed the parameter ${K}_{{\rm{SCS}}}$ to express the binding strength between a SCS and a MRS. In order to determine the averaged binding energy of a bacterium, Monte Carlo simulation^35^ was used assuming the dielectrophoreitic force by oscillating electric field with Equation (5). The energy variation by dielectrophretic force is given as $d{E}_{\sigma +1}=-{\sum }_{i} < {{\bf{F}}}_{i} > \cdot d{{\bf{r}}}_{i}$ after the random translational and rotational changes of a bacterium during the *σ*th and (*σ* + 1)th steps. If $d{E}_{\sigma +1}\le 0$ is satisfied, the (*σ* + 1)th state is always adopted since the energy of this state is lower than that of the *σ*th step. On the other hand, if $d{E}_{\sigma +1} > 0$ is satisfied, the (*σ* + 1)th state is adopted with the probability of $p=\exp (-d{E}_{\sigma +1}/{k}_{{\rm{B}}}T)$ or the configuration does not change since the energy of this state is higher than the *σ*th step. By repeating these procedures, we can sample the stochastic behavior of a bacterium.

## Electronic supplementary material

Movie S1

Movie S2

Movie S3

Supplementary information
