# Supplementary material for: Mechanism in External Field-mediated Trapping of Bacteria Sensitive to Nanoscale Surface Chemical Structure
Source: Sci Rep. 2017 Nov 30;7:16651. doi: 10.1038/s41598-017-15086-1 (PMC5709418; doi:10.1038/s41598-017-15086-1)
Supplement: Supplementary file 4 — Supplementary information [file 41598_2017_15086_MOESM4_ESM.pdf]

SUPPLEMENTARY INFORMATION

Mechanism in External Field-mediated Trapping of Bacteria Sensitive to Nanoscale Surface Chemical Structure

Shiho Tokonami[1,3,*], Emi Shimizu[1,3], Mamoru Tamura[2,3], and Takuya Iida[2,3,*]

[1]Department of Applied Chemistry, Graduate School of Engineering, Osaka Prefecture University, 1-2, Gakuencho, Nakaku, Sakai, Osaka 599-8570, Japan.
[2]Department of Physical Science, Graduate School of Science, Osaka Prefecture University, 1-2, Gakuencho, Nakaku, Sakai, Osaka 599-8570, Japan.
[3]Research Institute for Light-induced Acceleration System (RILACS), Osaka Prefecture University, 1-2 Gakuen-cho, Naka-ku, Sakai, Osaka 599-8570, Japan

*Correspondence to: t-iida@p.s.osakafu-u.ac.jp (T.I.), tokonami@chem.osakafu-u.ac.jp (S.T.);

This PDF file includes

Movie Legends (S1-S3)

Figures S1: Clausius-Mossotti factor related with polarizability of a bacterium

Figure S2: Simulation results of electric force on a bacterium.

Figure S3: Model of the surface chemical structure (SCS) and molecular recognition site (MRS).

Figure S4: Model of the electromagnetic boundary condition and environmental parameters in finite difference method (FDM) calculation for ring and disk electrode for Fig. 4c.

Movie Legends:

Please see also the detail in the main text.

Movie S1. Simulation result of configuration change of a Type 1 bacterium (mismatched) with a small number of SCS (291) under the electric field with electrical potential difference between the disk electrode and the ring electrode ΔV = 1.5 V. The spring constant of binding force between SCS and MRS was set to K_{rec}= 4 μN/m.

Movie S2. Simulation result of configuration change of a Type 2 bacterium (complementary) with a middle number of SCS (523) under the electric field with ΔV = 1.5 V. The spring constant of binding force between SCS and MRS was set to K_{rec}= 4 μN/m.

Movie S3. Simulation result of configuration change of a Type 3 bacterium (mismatched) with a large number of SCS (804) under the electric field with electrical potential difference between the disk electrode and the ring electrode ΔV = 1.5 V. The spring constant of binding force between SCS and MRS was set to K_{rec}= 4 μN/m.

Supplementary Figures:

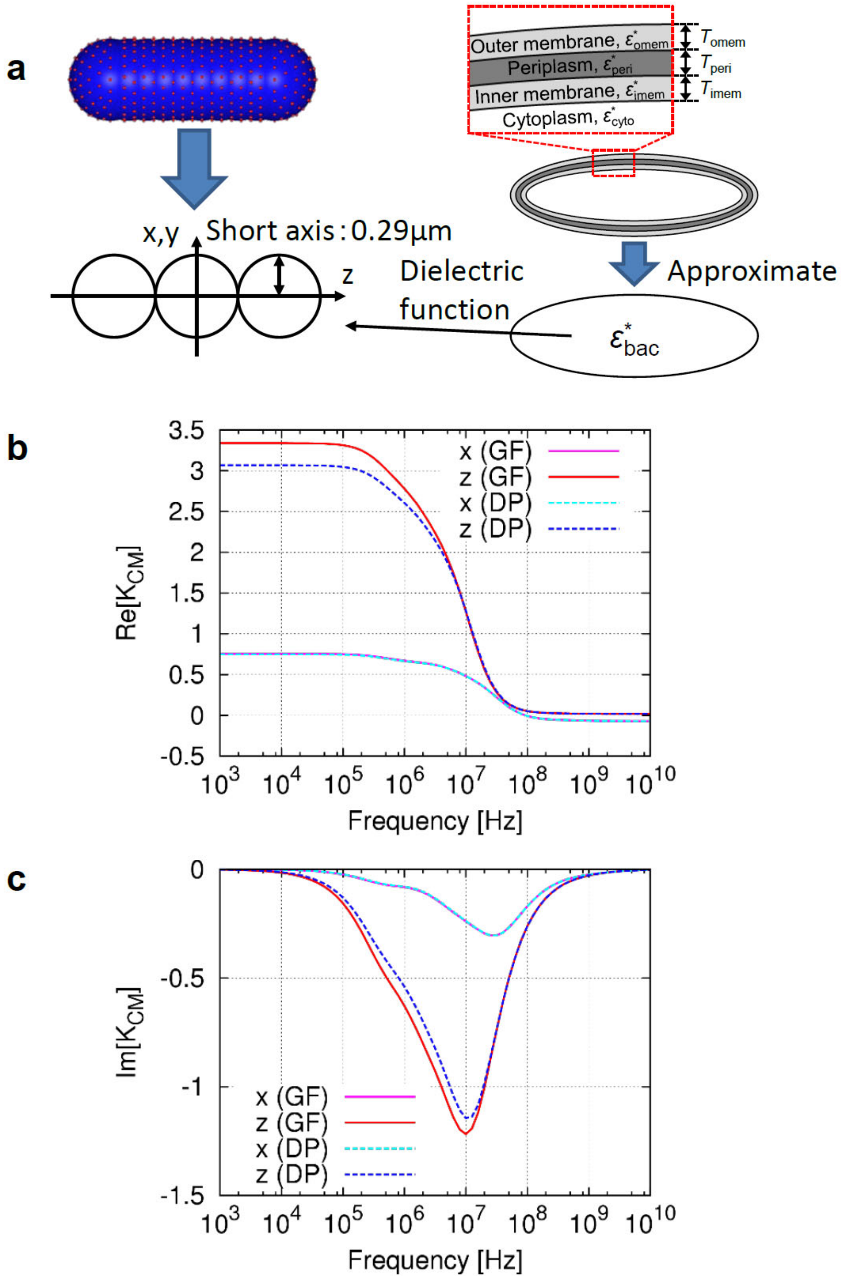

Fig. S1. Clausius-Mossotti factor related with polarizability of a bacterium ($K_{CM}(\omega)$). (a) Model for the calculation and outline of the approximation. (b) Real part of $K_{CM}(\omega)$ as a function of frequency of applied alternating electric field. (c) Imaginary part of $K_{CM}(\omega)$ as a function of frequency of applied alternating electric field. Results with GF were calculated by Green's function method, and results with DP were calculated by depolarization factor for spheroid with Clausius-Mossotti factor.

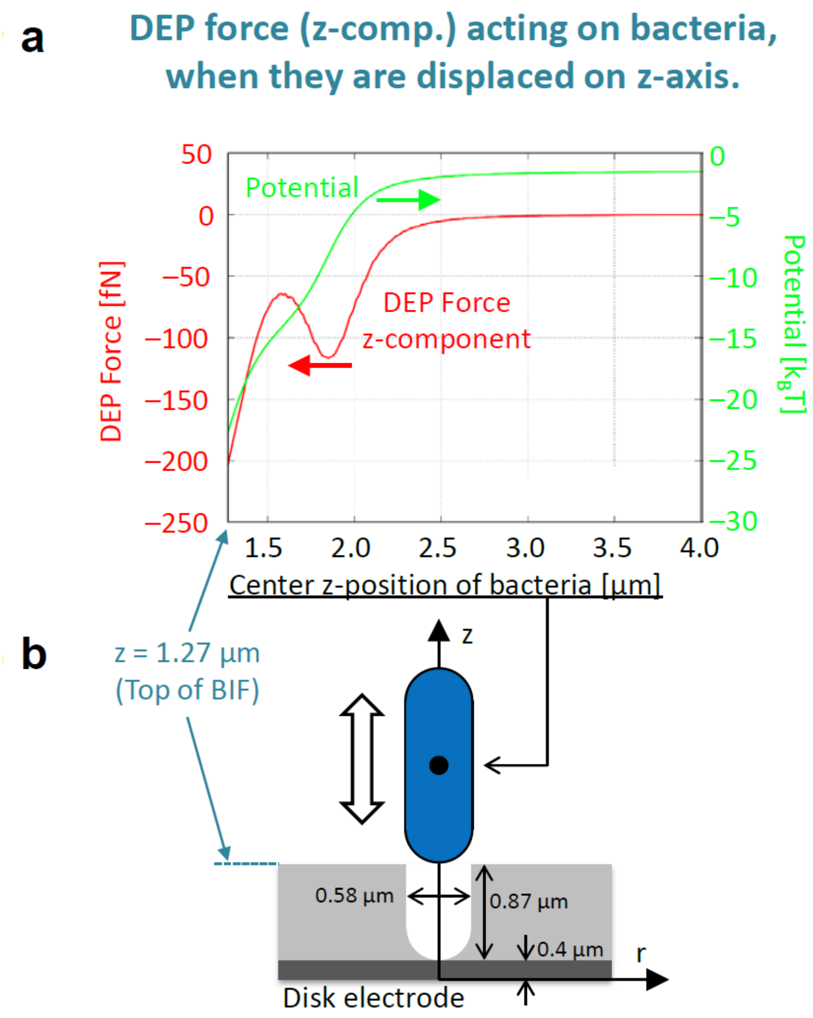

Fig. S2. Simulation results of electric force on a bacterium. (a) Dielectrophetic (DEP) force and corresponding potential as a function of the vertical position of a bacterium. (b) Model for the calculation of DEP force.

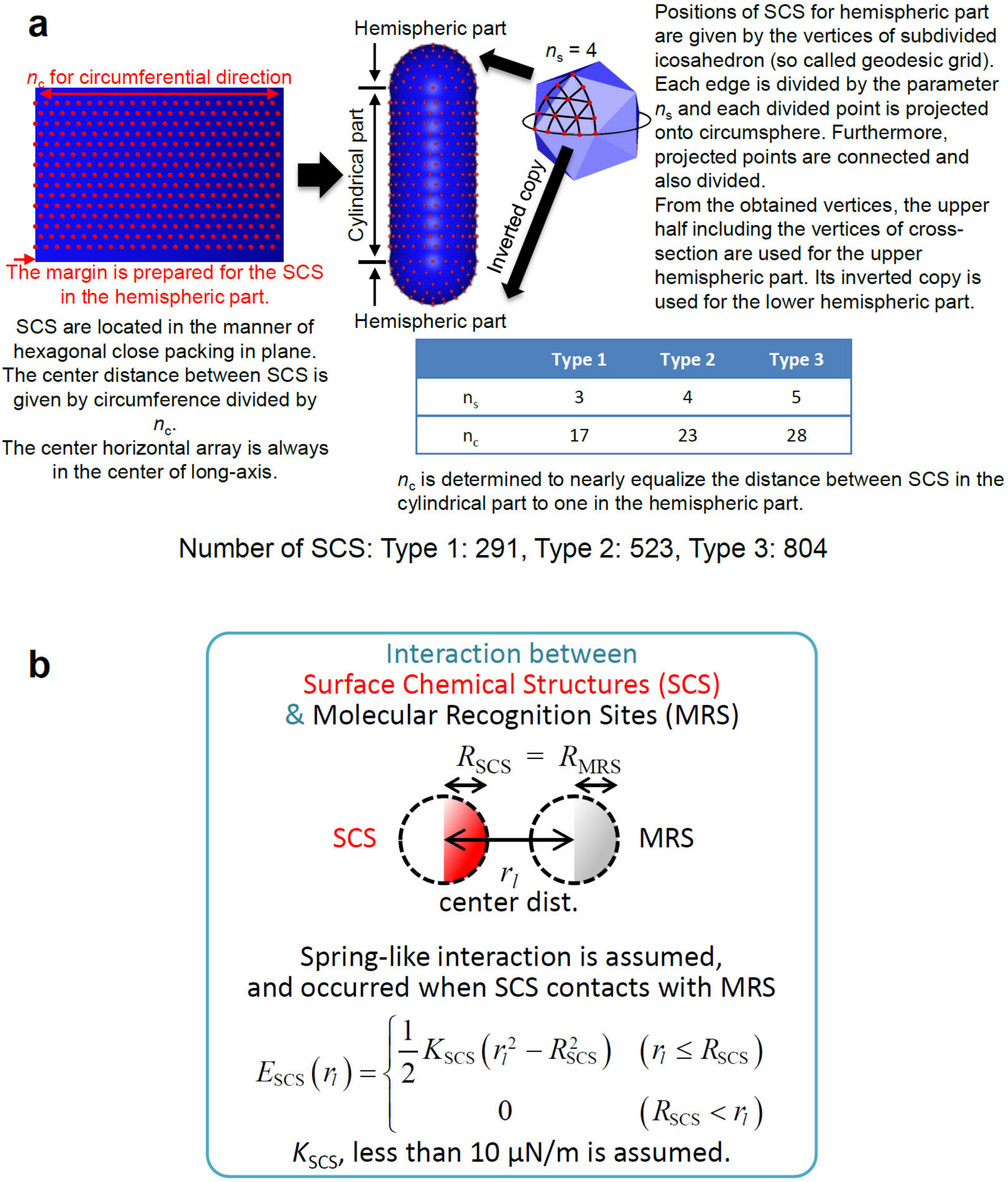

	Type 1	Type 2	Type 3
n_s	3	4	5
n_c	17	23	28

Fig. S3. Model of the surface chemical structure (SCS) and molecular recognition site (MRS). (a) Model for SCS of a bacterium. (b) Model for calculation of interaction between a pair of SCS and MRS.

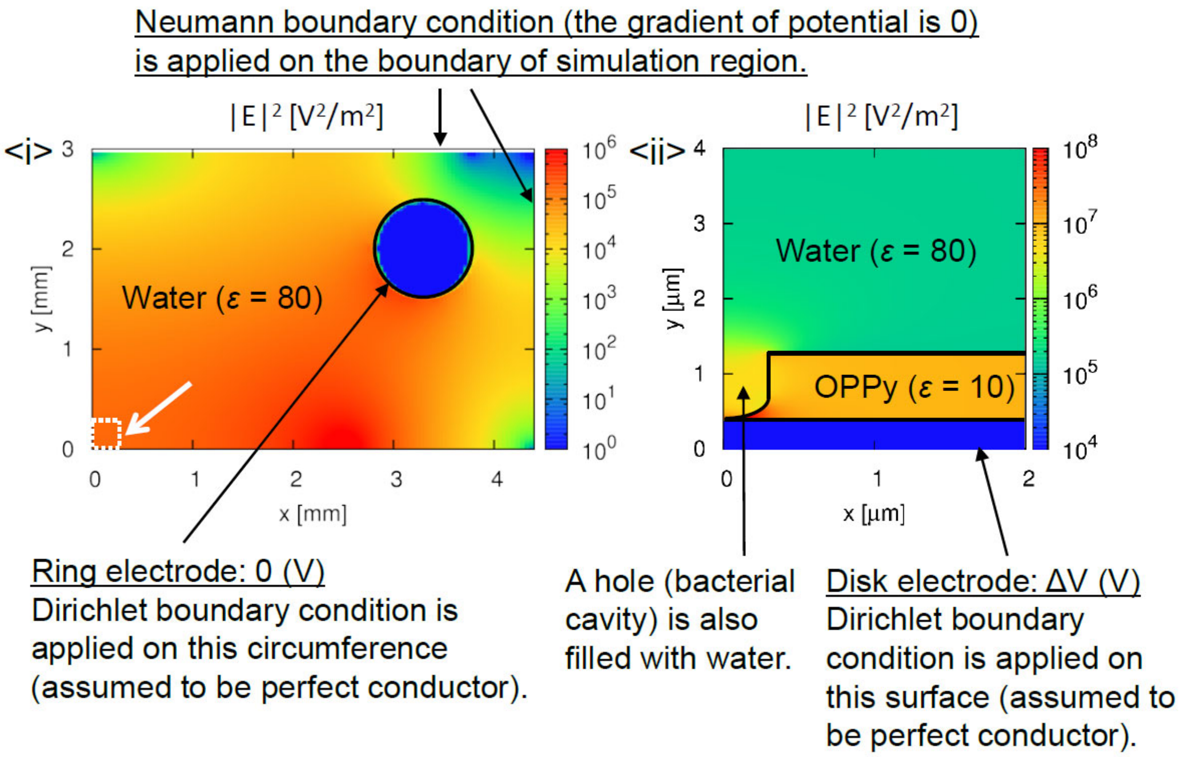

Fig. S4. Model of the electromagnetic boundary condition and environmental parameters in finite difference method (FDM) calculation for ring and disk electrode for Fig. 4c. <i> Spatial distribution of electric field around ring electrode and disk electrode, and <ii> the enlarged figure near the center of disk electrode (enclosed with white dotted square). The dielectric constant of OPPy was estimated suitable for the condition of dielectrophoretic trapping.
